# Methods for identifying 30 chronic conditions: application to administrative data

**DOI:** 10.1186/s12911-015-0155-5

**Published:** 2015-04-17

**Authors:** Marcello Tonelli, Natasha Wiebe, Martin Fortin, Bruce Guthrie, Brenda R Hemmelgarn, Matthew T James, Scott W Klarenbach, Richard Lewanczuk, Braden J Manns, Paul Ronksley, Peter Sargious, Sharon Straus, Hude Quan, For the Alberta Kidney Disease Network

**Affiliations:** Department of Medicine, University of Calgary, Calgary, Canada; Department of Medicine, University of Alberta, Edmonton, Canada; Department of Family Medicine, Université de Sherbrooke, Sherbrooke, Canada; Population Health Sciences Division, Medical Research Institute, University of Dundee, Dundee, UK; Alberta Health Services, Edmonton, Canada; Department of Community Health Sciences, University of Calgary, Calgary, Canada; Department of Medicine, University of Toronto, Toronto, Canada

**Keywords:** Multimorbidity, Administrative data

## Abstract

**Background:**

Multimorbidity is common and associated with poor clinical outcomes and high health care costs. Administrative data are a promising tool for studying the epidemiology of multimorbidity. Our goal was to derive and apply a new scheme for using administrative data to identify the presence of chronic conditions and multimorbidity.

**Methods:**

We identified validated algorithms that use ICD-9 CM/ICD-10 data to ascertain the presence or absence of 40 morbidities. Algorithms with both positive predictive value and sensitivity ≥70% were graded as “high validity”; those with positive predictive value ≥70% and sensitivity <70% were graded as “moderate validity”. To show proof of concept, we applied identified algorithms with high to moderate validity to inpatient and outpatient claims and utilization data from 574,409 people residing in Edmonton, Canada during the 2008/2009 fiscal year.

**Results:**

Of the 40 morbidities, we identified 30 that could be identified with high to moderate validity. Approximately one quarter of participants had identified multimorbidity (2 or more conditions), one quarter had a single identified morbidity and the remaining participants were not identified as having any of the 30 morbidities.

**Conclusions:**

We identified a panel of 30 chronic conditions that can be identified from administrative data using validated algorithms, facilitating the study and surveillance of multimorbidity. We encourage other groups to use this scheme, to facilitate comparisons between settings and jurisdictions.

**Electronic supplementary material:**

The online version of this article (doi:10.1186/s12911-015-0155-5) contains supplementary material, which is available to authorized users.

## Background

Management of chronic disease is the major challenge facing health systems worldwide [1]. Many people with chronic disease have multiple chronic conditions, which is termed multimorbidity [2]. It is clear that multimorbidity is common and associated with worse clinical outcomes and higher health care costs, compared to good health or to the presence of a single chronic condition [3-6]. However, there are key knowledge gaps concerning the basic epidemiology of multimorbidity [7]; its clinical and economic consequences; and how it contributes to disparities in health [8]. This information is prerequisite to mitigating the impact of multimorbidity and chronic disease [9].

Multimorbidity has been identified as a key research priority by the Public Health Agency of Canada, and is crucial to inform programming and resource forecasting. Knowledge of secular changes in the incidence and prevalence of multimorbidity is required, and would be facilitated by methods for identifying the presence of multimorbidity using administrative data.

Identifying morbidity using administrative data can be simple (e.g., based on a single hospitalization and using only a small number of codes) or complex (e.g., including inpatient and outpatient encounters and long lists of codes). Once developed, algorithms may be validated against a suitable gold standard (e.g., chart reviews; other previously validated algorithms).

Previous work by Barnett et al [3] identified 40 morbidities and were informed by a systematic review of multimorbidity measures [10], the Quality and Outcomes Framework of the UK General Practice contract, and health service planning by NHS Scotland. However, these authors used administrative data sources that are unique to the United Kingdom to identify the presence or absence of these conditions. A corresponding scheme based on the more widely used ICD-9 CM/ICD-10 system is not available.

Therefore, we first identified previously validated algorithms that use the ICD-9 CM/ICD-10 system for ascertaining the presence of chronic conditions using inpatient and outpatient claims and utilization data. We then showed proof of concept for using administrative data to study multimorbidity, by applying these previously validated algorithms to a population of adults residing in Edmonton, Canada between April 2008 and March 2009.

## Methods

The institutional review boards at the University of Alberta (Pro00038795) and the University of Calgary (E22590) approved the study.

### Morbidities

We did a focused literature search for validated algorithms that use ICD-9 CM/ICD-10 codes in administrative data from inpatient and outpatient encounters to ascertain the presence or absence of the 40 morbidities identified by Barnett et al [3]. We searched MEDLINE using combinations of the following MeSH subject headings together with the specific morbidity of interest: ‘International Classification of Diseases’, ‘Reproducibility of Results’, and ‘Sensitivity and Specificity’. Based on an a priori decision, we considered algorithms to be of high validity if they had both positive predictive value (PPV) and sensitivity ≥70% as compared to an acceptable gold standard such as chart review. We considered algorithms to be of moderate validity if they had PPV ≥70% but sensitivity <70%. The cut-off values for PPV and sensitivity were based on previous validation studies of administrative data [11]. We did not consider negative predictive value or specificity, because these parameters are generally >90% in studies of chronic diseases among the general population [11,12]. The definition of multimorbidity required the coexistence of two or more of the morbidities. In a secondary analysis we used a more restrictive definition that required three or more morbidities to be present.

### ICD codes

Canadian hospital discharge abstract data are coded with ICD-10 CA, which essentially increases specificity compared to the ICD-10 system by adding more digits [11]. All of the ICD-10 codes from the included algorithms are consistent with ICD-10 CA codes, and thus we used ICD-10 and ICD-10 CA codes interchangeably throughout this manuscript. When ICD-10 codes were not given in the primary papers, we used the Canadian Institute for Health Information (CIHI; www.cihi.ca) conversion table to convert ICD-9 CM codes to ICD-10 codes. Many algorithms required multiple codes within a specified time period to determine incidence of morbidity (Table 1). In each case the index date for the disease was considered to be the date of the first code. For example, in order to determine presence of asthma, we searched for ICD-9 CM 493 and ICD-10 J45 codes in hospitalizations and outpatient encounters. We considered asthma to have developed at the first instance of a single hospitalization with either of these codes, or a single outpatient encounter followed by two further outpatient encounters with either of these codes within two years. In either case, we considered the participant to have asthma for the duration of follow-up.

**Table 1:** **Administrative algorithms for 30 morbidities**

		**Number and nature of claims required to substantiate the diagnosis**							
**Morbidity**	**Algorithm**	**Hospitalizations**	**Claims**	**ACCS**	**Years**	**ICD-9 CM**	**ICD-10**	**Permanent**	**Citation**
Alcohol misuse^	1 hospitalization or 2 claims in 2 years or less	1	2	.	2	265.2, 291.1–291.3, 291.5–291.9, 303.0, 303.9, 305.0, 357.5, 425.5, 535.3, 571.0–571.3, 980, V11.3	E52, F10, G62.1, I42.6,K29.2, K70.0, K70.3, K70.9, T51, Z50.2, Z71.4, Z72.1	Y	Quan 2008 [11], 2005 [17]
Asthma	1 hospitalization or 3 ACCS in 2 years or less	1	.	3	2	493	J45	Y	Gershon 2009 [37]
Atrial fibrillation	1 hospitalization or 2 claims in 2 years or less	1	2	.	2	427.3^&^	I48.0	Y	Alonso 2009 [38]
Cancer, lymphoma	1 hospitalization or 2 claims in 2 years or less	1	2	.	2	200–202, 203.0, 238.6	C81–C85, C88, C90.0, C90.2, C96	N 5 y	Quan 2008 [11], 2005 [17]
Cancer, metastatic	1 hospitalization or 2 claims in 2 years or less	1	2	.	2	196–199	C77–C80	N 5 y	Quan 2008 [11], 2005 [17]
Cancer, non-metastatic (breast, cervical, colorectal, lung, prostate)	1 hospitalization or 2 claims in 2 years or less	1	2	.	2	153-154, 162-163, 174, 180, 185, 230.3-230.6, 231.2, 233.0-233.1, 233.4	C18-C21, C33-C34, C38.4, C45.0, C46.71, C50, C53, C61, D01.0-D01.3, D02.2, D05-D06, D07.5	N 5 y	Penberthy 2003 [39]
Chronic heart failure^$^^	1 hospitalization or 2 claims in 2 years or less	1	2	.	2	398.91, 402.01, 402.11, 402.91, 404.01, 404.03, 404.11, 404.13, 404.91, 404.93, 425.4–425.9, 428	I09.9, I25.5, I42.0, I42.5–I42.9, I43, I50	Y	Quan 2008 [11], 2005 [17]
Chronic kidney disease	Mean eGFR <60 mL/min*1.73 m^2^ or mean albuminuria >30 mg/g over 12 months	.	.	.	.	.	.		Stevens 2013 [16]
	or 1 hospitalization or 3 claims in 1 year	1	3	.	1	583, 584, 585, 586, 592, 593.9	N00-N23	Y	Ronksley 2012 [15]
Chronic pain	2 hospitalizations or 2 claims or 2 ACCS in 30 days or less	2	2	2	30 d	307.80, 307.89, 338.0, 338.2, 338.4, 719.41, 719.45 - 719.47, 719.49, 720.0, 720.2, 720.9, 721.0 - 721.4, 721.6, 721.8, 721.9, 722, 723.0, 723.1, 723.3 - 723.9, 724.0 - 724.6, 724.70, 724.79, 724.8, 724.9, 729.0 - 729.2, 729.4, 729.5	F45.4, M08.1, M25.50, M25.51, M25.55 - M25.57, M43.2 - M43.6, M45, M46.1, M46.3, M46.4, M46.9, M47, M48.0, M48.1, M48.8, M48.9, M50.8, M50.9, M51, M53.1 - M53.3, M53.8, M53.9, M54, M60.8, M60.9, M63.3, M79.0 - M79.2, M79.6, M79.7, M96.1	N 2 y	Tian 2013 [18]
Chronic pulmonary disease^%^	1 hospitalization or 2 claims in 2 years or less	1	2	.	2	416.8, 416.9, 490–492, 494-505, 506.4, 508.1, 508.8	I27.8, I27.9, J40–J44, J46-J47, J60–J67, J68.4, J70.1, J70.3	Y	Quan 2008 [11], 2005 [17]
Chronic viral hepatitis B	2 hospitalizations or 2 claims or 2 ACCS	2	2	2	6 mo	70.2-70.3	B16, B18.0-B18.1	Y	Mahajan 2013 [40]
Cirrhosis^	1 hospitalization or 1 claim or 1 ACCS	1	1	1	.	571.2, 571.5, 571.6	K70.3, K74.3, K74.4, K74.5, K74.6	Y	Goldberg 2012 [41]
	and hepatic decompensation 1 hospitalization or 1 claim or 1 ACCS	1	1	1	.	456.0, 456.1, 456.20, 456.21, 567.0, 567.2,* 567.21, 567.29, 567.8, 567.9, 572.2, 572.4, 789.5	I85.0, I85.9, I98.2, I98.3, K65.0, K65.8, K65.9, K67.0, K67.1, K67.2, K67.3, K67.8, K76.7, K93.0, R18		
						Exclude 567.81, 567.82, 789.51			
Dementia^	1 hospitalization or 2 claims in 2 years or less	1	2	.	2	290, 294.1, 331.2	F00–F03, F05.1, G30, G31.1	Y	Quan 2008 [11], 2005 [17]
Depression^	1 hospitalization or 2 claims in 2 years or less	1	2	.	2	296.2, 296.3, 296.5, 300.4, 309, 311	F20.4, F31.3–F31.5, F32, F33, F34.1, F41.2, F43.2	N 2 y	Quan 2008 [11], 2005 [17]
Diabetes	1 hospitalization or 2 claims in 2 years or less	1	2	.	2	250	E10-E14	Y	Hux 2005 [42]
Epilepsy	1 most responsible hospitalization or 2 claims in 2 years or less or 1 most responsible ACCS	1	2	1	2	345	G40-G41	Y	Jette 2010 [43]
Hypertension^	1 hospitalization or 2 claim in 2 years or less	1	2	.	2	401-405	I10-I13, I15	Y	Quan 2009 [44]
Hypothyroidism	1 hospitalization or 2 claims in 2 years or less	1	2	.	2	240.9, 243, 244, 246.1, 246.8	E00–E03, E89.0	Y	Quan 2008 [11], 2005 [17]
Inflammatory bowel disease	2 hospitalizations or 2 GAST or GP claims in 3 years or less	2	2	.	3	555, 556	K50, K51	Y	Liu 2009 [19]
Irritable bowel syndrome	1 hospitalization without surgery or 2 claims in 2 years or less	1	2	.	2	564.1	K58	Y	Sands 2006 [21]
						Exclude 153-154, 157, 183.0, 197.5, 198.6, 235.2, 239.0, 555-556, 571.2, 571.5, 577.1, 579	Exclude C18-C21, C25, C56, C78.5, C79.6, D01.7, D01.9, D37.1-D37.5, K50-K51, K70.2-K70.3, K74.0, K74.2, K74.6, K86.0-K86.1, K90, K91.2		
Multiple sclerosis	2 hospitalizations or 2 claims in 3 years or less	2	2	.	3	323, 340, 341.0, 341.9, 377.3	G35, G36, G37, H46	Y	Marrie 2013 [20]
Myocardial infarction	1 hospitalization	1	.	.	.	410	I21-I22	Y	Austin 2002 [45]
Parkinson’s disease	1 hospitalizations or 1 claim	1	1	.	.	332	G20, G21, G22	Y	Noyes 2007 [46]
Peptic ulcer disease	1 hospitalization or 2 claims in 2 years or less	1	2	.	2	531.7, 531.9, 532.7, 532.9, 533.7, 533.9, 534.7, 534.9	K25.7, K25.9, K26.7, K26.9, K27.7, K27.9, K28.7, K28.9	N 2 y	Quan 2008 [11], 2005 [17]
Peripheral vascular disease	1 hospitalization or 1 claim or 1 ACCS	1	1	1	.	440.2	I70.2	Y	Fan 2013 [47]
Psoriasis	1 hospitalization or 1 DERM claim	1	1	.	.	696.1	L40.0 - L40.4, L40.8, L40.9	Y	Asgari 2013 [48]
Rheumatoid arthritis	1 hospitalization or 2 claims in 2 years or less	1	2	.	2	446.5, 710.0–710.4, 714.0–714.2, 714.8, 725	M05, M06, M31.5, M32–M34, M35.1, M35.3, M36.0	Y	Quan 2008 [11], 2005 [17]
Schizophrenia^	1 hospitalization or 2 claims in 2 years or less	1	2	.	2	295	F20, F21, F23.2, F25	Y	Lurie 1992 [49], Moscovice 1989 [50]
Severe constipation	1 hospitalization without surgery or 2 claims in 2 years or less	1	2	.	2	560.1, 560.30, 560.39, 560.9, 564.0, 569.83, 569.89	K55.8, K56.0, K56.4, K56.7, K59.0, K63.1, K63.4, K63.81, K63.88, K92.80, K92.88	N 2 y	Sands 2006 [21]
						Exclude 152-154, 158, 179-189, 197.5-197.6, 235.2, 239.0, 555-556, 568.0, 614.6, (560.9 if 789.01, 789.02, 789.06), and any CCPx surgery^#^ listed in claims			
							Exclude C17-C21, C45.1, C48, C51-C58, C60-C68, C78.5-C78.6, D01.7, D01.9, D37.1-D37.5, K50-K51, K66.0, N73.6, N99.4 (K56.6 if R10.1), and any CCPx surgery^#^ listed in claims		
Stroke or TIA	1 most responsible or post-admittance hospitalization or 1 claim or 1 most responsible ED ACCS	1	1	1	.	362.3, 430, 431, 433.x1, 434.x1, 435, 436	G45.0-G45.3, G45.8-G45.9, H34.1, I60, I61, I63, I64	Y	Kokotailo 2005 [51]

ACCS ambulatory care classification system, ICD-9 CM International Classification of Diseases 9^th^ Revision Clinical Modification, ICD-10 International Classification of Diseases 10^th^ Revision, CCPx Canadian Classification of Procedures, eGFR estimated glomerular filtration rate, GAST gastroenterologist, GP general practitioner, DERM dermatologist, ED emergency department, TIA transient ischemic attack.

Any diagnosis field was considered in the algorithms except when the original text specified otherwise. The index date for disease was set as the date for the first relevant claim. Diseases denoted as “permanent” were assumed to be present continuously from the index date; diseases that are not “permanent” were considered to remit if no claims were present for a specified period of time.

*The author did not intend 567.2 to generalize and include 567.22 or 567.23

^#^CCPx surgery codes in claims were included as exclusions for hospitalizations for severe constipation.

^&^In claims the specific ICD-9 CM code for atrial fibrillation was not in use and so the less specific code 427.3 (which includes atrial flutter) was used in place of 427.31.

^%^The ICD-9 CM code 493 and the ICD-10 code J45 was removed from the chronic pulmonary disease algorithm as it was also included in the asthma algorithm.

^$^The ICD-10 codes I11 and I12 were eliminated from chronic heart failure as separate codes for heart failure are required.

^A number of codes were legitimately used in more than one algorithm: alcohol misuse and chronic heart failure (425.5, I42.6), alcohol misuse and cirrhosis (571.2, K70.3), chronic heart failure and hypertension (402.91, 404.01, 404.03, 404.11, 404.13, 404.91, 404.93), and depression and schizophrenia (F20.4).

### Proof of concept

**Figure 1:**
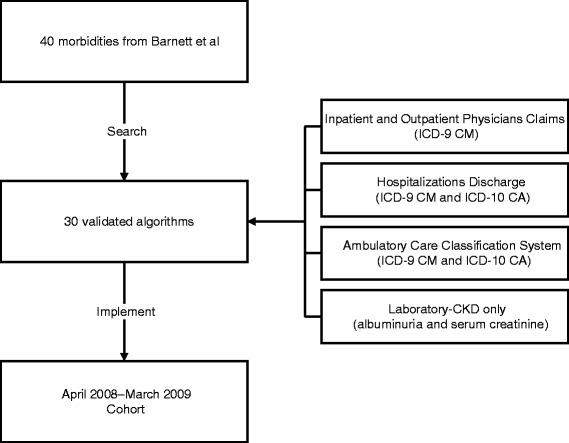
Development of the cohort. ICD International Classification for Diseases, CKD chronic kidney disease. Barnett K, Mercer SW, Norbury M, Watt G, Wyke S, Guthrie B. Epidemiology of multimorbidity and implications for health care, research, and medical education: a cross-sectional study. *Lancet.* 2012;380(9836):37-43.

We applied identified algorithms with high or moderate validity to a population-based administrative dataset from Alberta Health (AH; the provincial health ministry) and Alberta clinical laboratories. Details of this administrative dataset including claims, hospitalizations and Ambulatory Care Classification System (ACCS) utilization are given in Figure 1 and have been reported elsewhere [13]. We assembled a cohort of adults aged ≥18 years who resided in the city of Edmonton, Alberta between April 2008 and March 2009, and included all people registered with AH. All Alberta residents are eligible for insurance coverage by AH, and >99% participate in this coverage. The dataset included demographic information such as postal code of residence, laboratory data, and medication in those aged ≥65 years [13]. We identified Edmonton residents from the AH registry file using the community name variable from the Statistics Canada Postal Code 2008 Conversion file [14] (www.statcan.gc.ca).

To demonstrate proof of concept for applying these algorithms to a large administrative dataset, we presented a simple summary of the prevalence of morbidity and multimorbidity in the study population. Counts and percentages were presented along with a figure showing how the number of morbidities varies by age. In sensitivity analyses, we presented the prevalence of morbidity as assessed by different sources of administrative data.

## Results

### Algorithms

We identified 16 morbidities for which the best identified algorithm was of high validity: asthma, atrial fibrillation, metastatic cancer, chronic heart failure, chronic kidney disease, chronic pain, cirrhosis, diabetes, hypertension, irritable bowel syndrome, multiple sclerosis, myocardial infarction, peripheral vascular disease, psoriasis, schizophrenia and severe constipation. We identified an additional 14 morbidities (including two algorithms for other types of cancer and one algorithm for another type of liver disease) for which the best identified algorithm was of moderate validity: alcohol misuse, lymphoma, non-metastatic cancer (breast, cervical, colorectal, lung, and prostate), chronic pulmonary disease, chronic viral hepatitis B, dementia, depression, epilepsy, hypothyroidism, inflammatory bowel disease, Parkinson’s disease, peptic ulcer disease, rheumatoid arthritis, and stroke or transient ischemic attack. We excluded the remaining morbidities for which no suitable algorithm could be identified (Additional file 1 Table S1). Thus we identified 30 conditions using administrative algorithms (including ICD-9 CM and ICD-10 codes) that are summarized in the Table 1. Of these 30 algorithms, half were validated for both ICD-9 CM and ICD-10 codes.

We identified all conditions exclusively using ICD-9 CM and ICD-10 data with the exception of chronic kidney disease, for which we used a validated algorithm applied to ICD-9 CM and ICD-10 data [15] and supplemented using serum creatinine and albuminuria data [16]. We considered chronic kidney disease to be present if a participant met either the administrative or laboratory criteria.

In some cases, we made minor changes to the published algorithms to improve anticipated diagnostic performance, to increase consistency between algorithms used for the different conditions, and to include application to the outpatient setting. First, the original publications by Quan et al [11,17] required one hospitalization to identify the presence of a chronic condition; based on input from the first author of that paper, we modified this algorithm to allow either one inpatient code or two outpatient codes within two years to define the presence of these conditions. Second, to improve mapping of ICD-9 codes from the original (published) algorithm into ICD-10, we combined the ‘highly likely’ and the ‘likely’ codes from the original algorithm for chronic pain [18]. Third, for consistency, we modified algorithms that defined conditions as present if participants had two codes within any duration of follow-up (no matter how long) to require that the two codes occur within a three year period [19,20]. Fourth, we expanded the criteria for presence of atrial fibrillation, epilepsy, irritable bowel syndrome, and severe constipation to include two outpatient codes within two years for these conditions. However, to ensure that secondary (post-surgical) bowel complications were not incorrectly classified as chronic bowel conditions, we excluded any hospitalization for surgery when assessing the presence or absence of these conditions [21]. Fifth, we expanded the criteria for presence of stroke or TIA to include one outpatient code. Sixth, we reviewed all algorithms for overlapping codes (situations where the same code was used to identify more than one condition), and modified the algorithms to avoid double-counting of morbidities (see footnotes in the Table 1 for specific details).

### Application of the algorithms to the Edmonton cohort

The study cohort included 574,409 participants (Figure 1, Table 2). Almost two-thirds were less than 50 years of age. Ten percent were 70 years of age or older and the proportion of men and women was similar. Approximately half of all participants were not identified as having any of the 30 morbidities for which high or moderate validity algorithms existed. Approximately one quarter were identified as having one of these 30 morbidities. Another quarter were identified as having 2 or more of these 30 morbidities (meeting the primary criterion for multimorbidity), whereas 12% had three or more (meeting the secondary criterion for multimorbidity).

**Table 2:** **Morbidity characteristics of participants residing in Edmonton, Alberta during the April 2008 to March 2009 fiscal year**

	**Prevalence, %**				
**Characteristic**	**Final algorithm**	**Using hospitalizations, claims, and ACCS**	**Using only hospitalizations and claims**	**Using only hospitalizations and ACCS**	**Using only hospitalizations**
**Number of morbidities**					
One	23.3	24.0	23.9	12.0	6.9
Two	11.5	12.1	11.9	4.9	3.7
Three	5.6	6.0	5.8	2.5	2.1
Four	2.8	3.1	2.9	1.4	1.1
Five or more	3.4	3.7	3.4	1.8	1.4
**Morbidities**					
Alcohol misuse	2.4	2.8	**2.4**	2.4	1.8^a^
Asthma	2.3	6.9	6.8	**2.3** ^**b**^	2.0^b^
Atrial fibrillation	2.4	2.5	**2.4**	1.9	1.6^a^
Cancer, lymphoma	0.2	0.2	**0.2**	0.1^a^	0.1^a^
Cancer, metastatic	0.6	0.6	**0.6**	0.5	0.5
Cancer, non-metastatic	2.2	2.2	**2.2**	1.1^b^	1.0^b^
Chronic heart failure	2.5	2.6	**2.5**	1.6^a^	1.4^a^
Chronic kidney disease^1^	20.8	3.8 (20.9)	3.6 **(20.8)**	2.9 (20.4)	2.2 (20.1)^a^
Chronic pain	6.5	**6.5**	5.2	1.4^b^	0.1^b^
Chronic pulmonary disease	7.0	7.2	**7.0**	2.7^b^	2.3^b^
Chronic viral hepatitis B	0.1	**0.1**	0.1	0.1^b^	<0.1^b^
Cirrhosis	0.2	**0.2**	0.1	0.1	0.1^a^
Dementia	1.6	1.6	**1.6**	1.1^a^	0.9^a^
Depression	8.7	8.9	**8.7**	1.7^b^	1.0^b^
Diabetes	8.8	8.9	**8.8**	5.6^a^	3.1^b^
Epilepsy	1.4	**1.4**	1.2	0.7^b^	0.1^b^
Hypertension	22.8	23.0	**22.8**	8.8^b^	7.8^b^
Hypothyroidism	7.6	7.6	**7.6**	2.3^b^	2.2^b^
Inflammatory bowel disease	1.0	1.1	**1.0**	0.7^a^	0.2^b^
Irritable bowel syndrome	1.9	2.0	**1.9**	0.6^b^	0.4^b^
Multiple sclerosis	0.7	0.8	**0.7**	0.4^a^	0.1^b^
Myocardial infarction	1.7	-	-	-	**1.7**
Parkinson’s disease	0.5	0.5	**0.5**	0.3^b^	0.2^b^
Peptic ulcer disease	0.2	0.2	**0.2**	0.1^a^	0.1^b^
Peripheral vascular disease	0.7	**0.7**	0.6	0.3^b^	0.2^b^
Psoriasis	0.9	1.0	**0.9**	0.2^b^	0.1^b^
Rheumatoid arthritis	1.5	1.6	**1.5**	0.8^a^	0.5^b^
Schizophrenia	1.5	1.5	**1.5**	0.7^b^	0.6^b^
Severe constipation	0.6	0.7	**0.6**	0.4^a^	0.3^b^
Stroke or transient ischemic attack	4.2	**4.2**	3.8	2.4^a^	0.9^b^

^a^Indicate morbidities where removing a sources or sources resulted in prevalence reduced between 25 to 50% compared to estimates using all three sources. ^b^Demonstrates morbidities where prevalence was reduced >50% compared to all three sources.

The prevalence values in the first column and the bold prevalence values in the remaining columns are estimates from the final algorithms given in Table 1. The bolded prevalence values indicate which administrative datasets were used in the final algorithms.

^1^Values in brackets include laboratory data (estimated glomerular filtration rate and albuminuria) as gold standard measures of kidney function

The apparent prevalence of most morbidities was greatly reduced (often by 50% or more) when we assessed their presence or absence using hospitalization data only (Table 2). The addition of ACCS data to hospitalization and claims data made little difference to prevalence estimates, with the possible exceptions of chronic pain, hepatitis B and cirrhosis (the prevalences of which all changed by >20%). In most cases, the algorithms as originally validated resulted in a prevalence that was intermediate between the most inclusive approach (using hospitalization, claims and ACCS data) and the most restrictive approach (using hospitalization data only). As expected, adding gold standard laboratory data for kidney function (eGFR and albuminuria) resulted in substantial increases in the apparent prevalence of chronic kidney disease as compared to administrative data alone, regardless of which administrative data sources were used.

**Figure 2:**
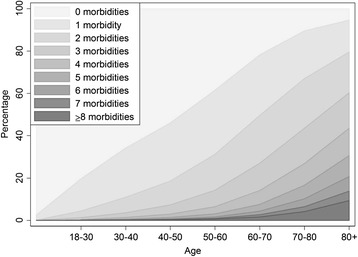
Number of morbidities by age in Edmonton, Alberta during the April 2008 to March 2009 fiscal year.

Figure 2 depicts the percentage of participants with multimorbidity by age group. After hypertension (a prevalence of 23%), 21% had chronic kidney disease, 9% had diabetes, and 9% had depression.

## Discussion

From a published list of chronic conditions [3], we identified a total of 30 validated algorithms including 3 algorithms for different types of cancer and 2 algorithms for liver disease. We applied the algorithms to ICD codes from claims and utilization data, and identified the presence or absence of these conditions in a cohort of 574,409 adults residing in Edmonton, Alberta between April 2008 and March 2009 (Figure 1). The overall prevalence of multimorbidity in this cohort was 26%, which is similar to the prevalence as reported in the Barnett study [3]. Our findings demonstrate proof of concept for using administrative data as a surveillance tool for multimorbidity in settings with systems for reliably capturing population-based claims and utilization data.

Multiple prior studies have ascertained the presence of various chronic conditions in the context of assessing multimorbidity [4,7,22-34]. Although there is no universally accepted definition of multimorbidity (or a list of conditions that should be used to assess the presence of multimorbidity) there appears to be consensus on several issues. First, health conditions used to define multimorbidity should be chronic but not necessarily permanent. Second, two or more concomitant conditions should be required to identify a person as having multimorbidity. Third, an attempt should be made to standardize definitions across studies to facilitate comparisons between populations [7,9,34,35]. At the same time, it is important that algorithms selected for use with administrative data should be validated against a gold standard – and demonstrate acceptable diagnostic properties so as to ensure reasonably accurate classification of individuals with respect to morbidity status. We focused on validated algorithms with positive predictive value and sensitivity ≥70%, compared to an acceptable gold standard such as chart review. Because we had access to laboratory data allowing a gold standard assessment of kidney function, we primarily assessed the presence of chronic kidney disease using estimated glomerular filtration rate (eGFR) and albuminuria rather than administrative data.

To our knowledge, this is the most comprehensive panel of validated algorithms yet applied to administrative data for the study of multimorbidity. Other studies have used reasonable but unvalidated algorithms, a more limited list of candidate chronic conditions or both. Although there are undoubtedly other chronic conditions that could be identified using administrative data, we focused on those for which available algorithms appear to have adequate sensitivity as well as positive predictive value. We will use the set of algorithms described herein as the foundation for a series of studies describing the epidemiology of multimorbidity in Alberta, Canada.

Besides the various definitions of multimorbidity that they have used, existing studies in this area have several other limitations [36]. First, population-based studies are rare (especially in Canadian settings); most studies have captured patients followed by a particular centre and are vulnerable to referral bias. Second, most studies have been unable to assess the link between multimorbidity and clinical outcomes in subgroups defined by age, sex, or low socioeconomic status. Third, little is known about the relative frequency of individual chronic conditions within the multimorbidity syndrome – or about which clusters of conditions are most common and/or clinically significant. Fourth, studies examining the economic consequences of multimorbidity have typically used relatively unsophisticated methods and/or studied only select populations. The scheme outlined in the current manuscript will allow our group to do future studies that close these knowledge gaps – informing policy and practice. We are optimistic that the scheme will also be used by other researchers from other jurisdictions with similar datasets – facilitating comparisons between studies. Future studies should test the relative importance of the morbidities identified in the current manuscript, as well as considering other potentially important morbidities for inclusion.

Limitations of the current approach include those common to all studies using administrative data. For example, we do not have information on potential confounders related to lifestyle (e.g., diet, smoking, exercise) or on measured blood pressure, which may be confounders when examining the association between multimorbidity and outcomes or costs. However, this limitation would not be expected to affect feasibility of applying the algorithms or the prevalence estimates reported here. Second, identification of some of the chronic conditions we studied might have been enhanced by simultaneous consideration of medication data [3]. We decided against including publicly funded medication data to define these conditions because medication coverage in Alberta is limited to people aged ≥65 years; those of lower SES; or with high annual medication costs. Thus, using medication data to define conditions would have biased towards a higher incidence of multimorbidity in older, poorer and sicker participants. We decided against restricting the cohort to people aged ≥65 years, because multimorbidity is relatively common in younger participants – and there might be important differences in the nature and implications of multimorbidity by age. Therefore, we will include all adult Albertans in our forthcoming analyses. Third, since participants must use medical services to be diagnosed with chronic conditions, our findings underestimate the true population burden of multimorbidity – especially for conditions that are less likely to lead to hospitalization but which may still significantly impact quality of life and other important outcomes. Fourth, we did not identify appropriate algorithms for all of the 40 target conditions, possibly because our searches were not exhaustive, and other important conditions such as obesity were not considered in this study. Therefore, our results likely underestimate the true prevalence of multimorbidity. Finally, although we focused on validated algorithms, the diagnostic performance of algorithms may vary between settings, based on coding practices and the reliability of data capture – and we did not systematically evaluate the quality of the original studies. Therefore (despite the lack of an *a priori* reason to suspect worse performance in our dataset), it is possible that some algorithms that were of high or moderate validity in other jurisdictions may perform less well when applied to Alberta data, especially with the modifications as described herein.

## Conclusions

In summary, we identified a panel of 30 chronic conditions that can be identified from administrative data using validated algorithms, facilitating the study and surveillance of multimorbidity. We encourage other groups to use this scheme, to facilitate comparisons of data on multimorbidity between settings and jurisdictions.

## Additional file

Additional file 1: Table S1. Validated algorithms for the original 40 morbidities.
