# Correction to: Methods for identifying 30 chronic conditions: application to administrative data

**DOI:** 10.1186/s12911-019-0900-2

**Published:** 2019-09-04

**Authors:** Marcello Tonelli, Natasha Wiebe, Martin Fortin, Bruce Guthrie, Brenda R. Hemmelgarn, Matthew T. James, Scott W. Klarenbach, Richard Lewanczuk, Braden J. Manns, Paul Ronksley, Peter Sargious, Sharon Straus, Hude Quan, For the Alberta Kidney Disease Network

**Affiliations:** 1 Department of Medicine, University of Calgary, Calgary, Canada; 2 Department of Medicine, University of Alberta, Edmonton, Canada; 3 Department of Family Medicine, Université de Sherbrooke, Sherbrooke, Canada; 4 Population Health Sciences Division, Medical Research Institute, University of Dundee, Dundee, UK; 5 Alberta Health Services, Edmonton, Canada; 6 Department of Community Health Sciences, University of Calgary, Calgary, Canada; 7 Department of Medicine, University of Toronto, Toronto, Canada

**Correction to: BMC Med Inform Decis Mak**

**http://dx.doi.org/10.1186/s12911-015-0155-5**

Following publication of the original manuscript [1], the authors noted several errors in Table 1.

**Table 1:** Administrative algorithms for 30 morbidities

		Number and nature of claims required to substantiate the diagnosis							
Morbidity	Algorithm	Hospitalizations	Claims	ACCS	Years	ICD-9 CM	ICD-10	Permanent	Citation
Alcohol misuse^	1 hospitalization or 2 claims in 2 years or less	1	2	.	2	265.2, 291.1–291.3, 291.5–291.9, 303.0, 303.9, 305.0, 357.5, 425.5, 535.3, 571.0–571.3, 980, V11.3	E52, F10, G62.1, I42.6,K29.2, K70.0, K70.3, K70.9, T51, Z50.2, Z71.4, Z72.1	Y	Quan 2008 [11], 2005 [17]
Asthma	1 hospitalization or 3 ACCS in 2 years or less	1	.	3	2	493	J45	Y	Gershon 2009 [37]
Atrial fibrillation	1 hospitalization or 2 claims in 2 years or less	1	2	.	2	427.3^&^	I48.0	Y	Alonso 2009 [38]
Cancer, lymphoma	1 hospitalization or 2 claims in 2 years or less	1	2	.	2	200–202, 203.0, 238.6	C81–C85, C88, C90.0, C90.2, C96	N 5 y	Quan 2008 [11], 2005 [17]
Cancer, metastatic	1 hospitalization or 2 claims in 2 years or less	1	2	.	2	196–199	C77–C80	N 5 y	Quan 2008 [11], 2005 [17]
Cancer, non-metastatic (breast, cervical, colorectal, lung, prostate)	1 hospitalization or 2 claims in 2 years or less	1	2	.	2	153–154, 162–163, 174, 180, 185, 230.3–230.6, 231.2, 233.0–233.1, 233.4	C18-C21, C33-C34, C38.4, C45.0, C46.71, C50, C53, C61, D01.0-D01.3, D02.2, D05-D06, D07.5	N 5 y	Penberthy 2003 [39]
Chronic heart failure^$^^	1 hospitalization or 2 claims in 2 years or less	1	2	.	2	398.91, 402.01, 402.11, 402.91, 404.01, 404.03, 404.11, 404.13, 404.91, 404.93, 425.4–425.9, 428	I09.9, I25.5, I42.0, I42.5–I42.9, I43, I50	Y	Quan 2008 [11], 2005 [17]
	Mean eGFR < 60 mL/min*1.73m^2^ or mean albuminuria > 30 mg/g over 12 months	.	.	.	.	.	.		Stevens 2013 [16]
Chronic kidney disease	or 1 hospitalization or 3 claims in 1 year	1	3	.	1	583, 584, 585, 586, 592, 593.9	N00-N23	Y	Ronksley 2012 [15]
Chronic pain	2 hospitalizations or 2 claims or 2 ACCS in 30 days or **more**	2	2	2	30 d	307.80, 307.89, 338.0, 338.2, 338.4, 719.41, 719.45, 719.46, 719.47, 719.49, 720.0, 720.2, 720.9, 721.0, 721.1, 721.2, 721.3, 721.4, 721.6, 721.8, 721.9, 722, 723.0, 723.1, 723.3, 723.4, 723.5, 723.6, 723.7, 723.8, 723.9, 724.0, 724.1, 724.2, 724.3, 724.4, 724.5, 724.6, 724.70, 724.79, 724.8, 724.9, 729.0, 729.1, 729.2, 729.4, 729.5	F45.4, **G89.0, G89.2, G89.4**, M08.1, M25.50, M25.51, M25.55, M25.56, M25.57, M43.2, M43.3, M43.4, M43.5, M43.6, M45, M46.1, M46.3, M46.4, M46.9, M47, M48.0, M48.1, M48.8, M48.9, M50.8, M50.9, M51, M53.1, M53.2, M53.3, M53.8, M53.9, M54, M60.8, M60.9, M63.3, M79.0, M79.1, M79.2, M79.6, M79.7, M96.1	N 2 y	Tian 2013 [18]
Chronic pulmonary disease^%^	1 hospitalization or 2 claims in 2 years or less	1	2	.	2	416.8, 416.9, 490–492, 494–505, 506.4, 508.1, 508.8	I27.8, I27.9, J40–J44, J46-J47, J60–J67, J68.4, J70.1, J70.3	Y	Quan 2008 [11], 2005 [17]
Chronic viral hepatitis B	2 hospitalizations or 2 claims or 2 ACCS	2	2	2	6 mo	70.2–70.3	B16, B18.0-B18.1	Y	Mahajan 2013 [40]
	1 hospitalization or 1 claim or 1 ACCS	1	1	1	.	571.2, 571.5, 571.6	K70.3, K74.3, K74.4, K74.5, K74.6		
Cirrhosis^	and hepatic decompensation 1 hospitalization or 1 claim or 1 ACCS	1	1	1	.	456.0, 456.1, 456.20, 456.21, 567.0, 567.2,* 567.21, 567.29, 567.8, 567.9, 572.2, 572.4, 789.5 Exclude 567.81, 567.82, 789.51	I85.0, I85.9, I98.2, I98.3, K65.0, K65.8, K65.9, K67.0, K67.1, K67.2, K67.3, K67.8, K76.7, K93.0, R18	Y	Goldberg 2012 [41]
Dementia^	1 hospitalization or 2 claims in 2 years or less	1	2	.	2	290, 294.1, 331.2	F00–F03, F05.1, G30, G31.1	Y	Quan 2008 [11], 2005 [17]
Depression^	1 hospitalization or 2 claims in 2 years or less	1	2	.	2	296.2, 296.3, 296.5, 300.4, 309, 311	F20.4, F31.3–F31.5, F32, F33, F34.1, F41.2, F43.2	N 2 y	Quan 2008 [11], 2005 [17]
Diabetes	1 hospitalization or 2 claims in 2 years or less	1	2	.	2	250	E10-E14	Y	Hux **2002** [42]
Epilepsy	1 most responsible hospitalization or 2 claims in 2 years or less or 1 most responsible ACCS	1	2	1	2	345	G40-G41	Y	Jette 2010 [43]
Hypertension^	1 hospitalization or 2 claim in 2 years or less	1	2	.	2	401–405	I10-I13, I15	Y	Quan 2009 [44]
Hypothyroidism	1 hospitalization or 2 claims in 2 years or less	1	2	.	2	240.9, 243, 244, 246.1, 246.8	E00–E03, E89.0	Y	Quan 2008 [11], 2005 [17]
Inflammatory bowel disease	2 hospitalizations or 2 GAST or GP claims in 3 years or less	2	2	.	3	555, 556	K50, K51	Y	Liu 2009 [19]
Irritable bowel syndrome	1 hospitalization without surgery or 2 claims in 2 years or less	1	2	.	2	564.1 Exclude 153–154, 157, 183.0, 197.5, 198.6, 235.2, 239.0, 555–556, 571.2, 571.5, 577.1, 579	K58 Exclude C18-C21, C25, C56, C78.5, C79.6, D01.7, D01.9, D37.1-D37.5, K50-K51, K70.2-K70.3, K74.0, K74.2, K74.6, K86.0-K86.1, K90, K91.2	Y	Sands 2006 [21]
Multiple sclerosis	2 hospitalizations or 2 claims in 3 years or less	2	2	.	3	323, 340, 341.0, 341.9, 377.3	G35, G36, G37, H46	Y	Marrie 2013 [20]
Myocardial infarction	1 **most responsible** hospitalization	1	.	.	.	410	I21-I22	Y	Austin 2002 [45]
Parkinson’s disease	1 hospitalizations or 1 claim	1	1	.	.	332	G20, G21, G22	Y	Noyes 2007 [46]
Peptic ulcer disease	1 hospitalization or 2 claims in 2 years or less	1	2	.	2	531.7, 531.9, 532.7, 532.9, 533.7, 533.9, 534.7, 534.9	K25.7, K25.9, K26.7, K26.9, K27.7, K27.9, K28.7, K28.9	N 2 y	Quan 2008 [11], 2005 [17]
Peripheral vascular disease	1 hospitalization or 1 claim or 1 ACCS	1	1	1	.	440.2	I70.2	Y	Fan 2013 [47]
Psoriasis	1 hospitalization or 1 DERM claim	1	1	.	.	696.1	L40.0, L40.1, L40.2, L40.3, L40.4, L40.8, L40.9	Y	Asgari 2013 [48]
Rheumatoid arthritis	1 hospitalization or 2 claims in 2 years or less	1	2	.	2	446.5, 710.0–710.4, 714.0–714.2, 714.8, 725	M05, M06, M31.5, M32–M34, M35.1, M35.3, M36.0	Y	Quan 2008 [11], 2005 [17]
Schizophrenia^	1 hospitalization or 2 claims in 2 years or less	1	2	.	2	295	F20, F21, F23.2, F25	Y	Lurie 1992 [49], Moscovice 1989 [50]
Severe constipation	1 hospitalization without surgery or 2 claims in 2 years or less	1	2	.	2	560.1, 560.30, 560.39, 560.9, 564.0, 569.83, 569.89 Exclude 152–154, 158, 179–189, 197.5–197.6, 235.2, 239.0, 555–556, 568.0, 614.6, (560.9 if 789.01, 789.02, 789.06), and any CCPx surgery^#^ listed in claims	K55.8, K56.0, K56.4, K56.7, K59.0, K63.1, K63.4, K63.81, K63.88, K92.80, K92.88 Exclude C17-C21, C45.1, C48, C51-C58, C60-C68, C78.5-C78.6, D01.7, D01.9, D37.1-D37.5, K50-K51, K66.0, N73.6, N99.4 (K56.6 if R10.1), and any CCPx surgery^#^ listed in claims	N 2 y	Sands 2006 [21]
Stroke or TIA	1 most responsible or post-admittance hospitalization or 1 claim or 1 most responsible ED ACCS	1	1	1	.	362.3, 430, 431, 433.×1, 434.×1, 435, 436	G45.0-G45.3, G45.8-G45.9, H34.1, I60, I61, I63, I64	Y	Kokotailo 2005 [51]

ACCS ambulatory care classification system, ICD-9 CM International Classification of Diseases 9th Revision Clinical Modification, ICD-10 International Classification of Diseases 10th Revision, CCPx Canadian Classification of Procedures, eGFR estimated glomerular filtration rate, GAST gastroenterologist, GP general practitioner, DERM dermatologist, ED emergency department

Any diagnosis field was considered in the algorithms except when the original text specified otherwise. The index date for disease was set as the date for the first relevant claim. Diseases denoted as “permanent” were assumed to be present continuously from the index date; diseases that are not “permanent” were considered to remit if no claims were present for a specified period of time

*The author did not intend 567.2 to generalize and include 567.22 or 567.23

^#^CCPx surgery codes in claims were included as exclusions for hospitalizations for severe constipation

^&^In claims the specific ICD-9 CM code for atrial fibrillation was not in use and so the less specific code 427.3 (which includes atrial flutter) was used in place of 427.31

^%^The ICD-9 CM code 493 and the ICD-10 code J45 was removed from the chronic pulmonary disease algorithm as it was also included in the asthma

^$^The ICD-10 codes I11 and I12 were eliminated from chronic heart failure as separate codes for heart failure are required

^A number of codes were legitimately used in more than one algorithm: alcohol misuse and chronic heart failure (425.5, I42.6), alcohol misuse and cirrhosis (571.2, K70.3), chronic heart failure and hypertension (402.91, 404.01, 404.03, 404.11, 404.13, 404.91, 404.93), and depression and schizophrenia (F20.4)

Details of the requested corrections are shown below:

1. Chronic pain – added 3 ICD-10 codes to the far right column that are equivalent to the 3 corresponding codes in the ICD-9 column. Neither the ICD-9 nor the ICD-10 codes are used in Canadian data, but we have corrected this for completeness. We thank Mr. James Wilton for pointing this out.

- G89.0 for 338.0 central pain syndrome
- G89.2 for 338.2 chronic pain
- G89.4 for 338.4 chronic pain syndrome

2. Also for chronic pain – replaced 2 hospitalizations or 2 claims or 2 ACCS in 30 days or **less** with 2 hospitalizations or 2 claims or 2 ACCS in 30 days or **more.** We thank Ms. Feng Ye for pointing this out.
3. For Diabetes – replaced the reference to Hux **2005** with a reference to Hux **2002.**
4. Myocardial infarction – replaced “1 hospitalization” with “1 **most responsible** hospitalization”. We thank Dr. Ping Liu for pointing this out.

The correct version of Table 1 is included in this erratum with the changes marked in bold.
