# Supplementary material for: Methods for identifying 30 chronic conditions: application to administrative data
Source: BMC Med Inform Decis Mak. 2015 Apr 17;15:31. doi: 10.1186/s12911-015-0155-5 (PMC4415341; doi:10.1186/s12911-015-0155-5)
Supplement: Additional file 1: Table S1. — Validated algorithms for the original 40 morbidities. [file 12911_2015_155_MOESM1_ESM.docx]

**Supplemental Table. Validated algorithms for the original 40 morbidities***

| **Morbidity** | **Validity** | **High**  **validity** | **Moderate**  **validity** | **Excluded** |
| --- | --- | --- | --- | --- |
| Alcohol misuse | Moderate (PPV 83% / Sn 54%) ICD-9 CM  Moderate (PPV 84% / Sn 52%) ICD-10 [[1](#_ENREF_1)] |  | X |  |
| Anorexia or bulimia | Low (PPV na / Sn na) |  |  | X |
| Anxiety disorders | Low (PPV na / Sn na) |  |  | X |
| Asthma | High (PPV 72% / Sn 74%) ICD-9 CM [[2](#_ENREF_2)] | X | X |  |
| Atrial fibrillation | High (PPV 89% / Sn 84%) ICD-9 CM [[3](#_ENREF_3)] | X | X |  |
| Blindness | Low (PPV na / Sn na) |  |  | X |
| Bronchiectasis | Low (PPV na / Sn na) |  |  | X |
| Cancer, lymphoma | Moderate (PPV 73% / Sn 66%) ICD-9 CM  Moderate (PPV 79% / Sn 63%) ICD-10 [[1](#_ENREF_1)] |  | X |  |
| Cancer, metastatic | High (PPV 89% / Sn 83%) ICD-9 CM  High (PPV 87% / Sn 81%) ICD-10 [[1](#_ENREF_1)] | X | X |  |
| Cancer, non-metastatic (breast, cervical, colorectal, lung, prostate) | Moderate (PPV 88% / Sn 62%) ICD-9 CM [[4](#_ENREF_4)] |  | X |  |
| Chronic heart failure | High (PPV 72% / Sn 91%) ICD-9 CM  Low (PPV 69% / Sn 90%) ICD-10 [[1](#_ENREF_1)] | X | X |  |
| Chronic kidney disease^&^ | High (using eGFR and Alb) [[5-7](#_ENREF_5)]  Low (PPV 64% / Sn 12%) ICD-9 CM/ ICD-10 [[8](#_ENREF_8)] | X | X |  |
| Chronic pain | High (PPV 95% / Sn 71%) ICD-9 CM [[9](#_ENREF_9)] | X | X |  |
| Chronic pulmonary disease | Moderate (PPV 92% / Sn 55%) ICD-9 CM  Moderate (PPV 91% / Sn 53%) ICD-10 [[1](#_ENREF_1)] |  | X |  |
| Chronic sinusitis | Low (PPV na / Sn na) |  |  | X |
| Chronic viral hepatitis B | Moderate (PPV 90% / Sn 58%) ICD-9 CM [[10](#_ENREF_10)] |  | X |  |
| Cirrhosis | High (PPV 86% / Sn 89%) ICD-9 CM [[11](#_ENREF_11)] | X | X |  |
| Dementia | Moderate (PPV 96% / Sn 32%) ICD-9 CM  Moderate (PPV 93% / Sn 67%) ICD-10 [[1](#_ENREF_1)] |  | X |  |
| Depression | Moderate (PPV 80% / Sn 57) ICD-9 CM  Moderate (PPV 92% / Sn 45%) ICD-10 [[1](#_ENREF_1)] |  | X |  |
| Diabetes | High (PPV 80% / Sn 86%) ICD-9 CM [[12](#_ENREF_12)] | X | X |  |
| Diverticulosis | Low (PPV na / Sn na) |  |  | X |
| Dyspepsia | Low (PPV na / Sn na) |  |  | X |
| Epilepsy | Moderate (PPV 99% / Sn na) ICD-9 CM  Moderate (PPV 99% / Sn na) ICD-10 [[13](#_ENREF_13)] |  | X |  |
| Glaucoma | Low (PPV na / Sn 75%) ICD-9 CM [[14](#_ENREF_14)] |  |  | X |
| Hearing loss | Low (PPV na / Sn na) |  |  | X |
| Hypertension | High (PPV 95% / Sn 79%) ICD-9 CM  Moderate (PPV 93% / Sn 68%) ICD-10 [[1](#_ENREF_1)] | X | X |  |
| Hypothyroidism | Moderate (PPV 93% / Sn 65%) ICD-9 CM  Moderate (PPV 93% / Sn 39%) ICD-10 [[1](#_ENREF_1)] |  | X |  |
| Inflammatory bowel disease | Moderate (PPV 95% / Sn na) ICD-9 CM [[15](#_ENREF_15)] |  | X |  |
| Irritable bowel syndrome | High (PPV 91% / Sn 99%) ICD-9 CM [[16](#_ENREF_16)] | X | X |  |
| Learning disability | Low (PPV na / Sn na) |  |  | X |
| Migraine | Low (PPV na / Sn na) |  |  | X |
| Multiple sclerosis | High (PPV 93% / Sn 91%) ICD-9 CM/ ICD-10 [[17](#_ENREF_17)] | X | X |  |
| Myocardial infarction | High (PPV 89% / Sn 89%) ICD-9 CM [[18](#_ENREF_18)] | X | X |  |
| Non-alcohol drug misuse | Low (PPV na / Sn na) |  |  | X |
| Parkinson’s disease | Moderate (PPV 79% / Sn 49%) ICD-9 CM [[19](#_ENREF_19)] |  | X |  |
| Peptic ulcer disease | Moderate (PPV 84% / Sn 37%) ICD-9 CM  Moderate (PPV 77% / Sn 40%) ICD-10 [[1](#_ENREF_1)] |  | X |  |
| Peripheral vascular disease | High (PPV 94% / Sn 77%) ICD-9 CM [[20](#_ENREF_20)] | X | X |  |
| Prostate disorders | Low (PPV na / Sn na) |  |  | X |
| Psoriasis | High (PPV 89% / Sn 91%) ICD-9 CM [[21](#_ENREF_21)] | X | X |  |
| Rheumatoid arthritis | Moderate (PPV 90% / Sn 51%) ICD-9 CM  Moderate (PPV 97% / Sn 53%) ICD-10 [[1](#_ENREF_1)] |  | X |  |
| Schizophrenia | High (PPV 87% / Sn 87%) ICD-9 CM [[22](#_ENREF_22), [23](#_ENREF_23)] | X | X |  |
| Severe constipation | High (PPV 73% / Sn 80%) ICD-9 CM [[16](#_ENREF_16)] | X | X |  |
| Stroke or TIA | Moderate (PPV 90% / Sn na) ICD-9 CM  Moderate (PPV 92% / Sn na) ICD-10 [[24](#_ENREF_24)] |  | X |  |
| **TOTAL NUMBER OF MORBIDITIES** |  | **16** | **30** | **13** |

PPV positive predictive value, Sn sensitivity, na not available

*Of the original set of 40 morbidities, we identified 30 algorithms to identify a total of 27 conditions (3 algorithms were used to identify cancer and 2 algorithms were used to identify liver disease).

^&^Chronic kidney disease was included although the administrative algorithm alone does not meet validity requirements. Serum creatinine and albuminuria data were used in addition to the administrative algorithm.

^#^All the ICD-10 codes present in this manuscript are consistent with ICD-10 CA codes

References

1. Quan H, Li B, Saunders LD, Parsons GA, Nilsson CI, Alibhai A, Ghali WA, Imecchi Investigators: **Assessing validity of ICD-9-CM and ICD-10 administrative data in recording clinical conditions in a unique dually coded database**. *Health Serv Res* 2008, **43**(4):1424-1441.

2. Gershon AS, Wang C, Guan J, Vasilevska-Ristovska J, Cicutto L, To T: **Identifying patients with physician-diagnosed asthma in health administrative databases**. *Can Respir J* 2009, **16**(6):183-188.

3. Alonso A, Agarwal SK, Soliman EZ, Ambrose M, Chamberlain AM, Prineas RJ, Folsom AR: **Incidence of atrial fibrillation in whites and African-Americans: the Atherosclerosis Risk in Communities (ARIC) study**. *Am Heart J* 2009, **158**(1):111-117.

4. Penberthy L, McClish D, Pugh A, Smith W, Manning C, Retchin S: **Using hospital discharge files to enhance cancer surveillance**. *Am J Epidemiol* 2003, **158**(1):27-34.

5. Hemmelgarn BR, Clement F, Manns BJ, Klarenbach S, James MT, Ravani P, Pannu N, Ahmed SB, MacRae J, Scott-Douglas N *et al*: **Overview of the Alberta Kidney Disease Network**. *BMC Nephrol* 2009, **10**:30.

6. James MT, Hemmelgarn BR, Wiebe N, Pannu N, Manns BJ, Klarenbach SW, Tonelli M, Alberta Kidney Disease N: **Glomerular filtration rate, proteinuria, and the incidence and consequences of acute kidney injury: a cohort study**. *Lancet* 2010, **376**(9758):2096-2103.

7. Stevens PE, Levin A, Kidney Disease: Improving Global Outcomes Chronic Kidney Disease Guideline Development Work Group Members: **Evaluation and management of chronic kidney disease: synopsis of the kidney disease: improving global outcomes 2012 clinical practice guideline**. *Ann Intern Med* 2013, **158**(11):825-830.

8. Ronksley PE, Tonelli M, Quan H, Manns BJ, James MT, Clement FM, Samuel S, Quinn RR, Ravani P, Brar SS *et al*: **Validating a case definition for chronic kidney disease using administrative data**. *Nephrol Dial Transplant* 2012, **27**(5):1826-1831.

9. Tian TY, Zlateva I, Anderson DR: **Using electronic health records data to identify patients with chronic pain in a primary care setting**. *J Am Med Inform Assoc* 2013.

10. Mahajan R, Moorman AC, Liu SJ, Rupp L, Klevens RM, Chronic Hepatitis Cohort Study investigators: **Use of the International Classification of Diseases, 9th revision, coding in identifying chronic hepatitis B virus infection in health system data: implications for national surveillance**. *J Am Med Inform Assoc* 2013, **20**(3):441-445.

11. Goldberg D, Lewis J, Halpern S, Weiner M, Lo Re V, 3rd.: **Validation of three coding algorithms to identify patients with end-stage liver disease in an administrative database**. *Pharmacoepidemiol Drug Saf* 2012, **21**(7):765-769.

12. Hux JE, Ivis F, Flintoft V, Bica A: **Diabetes in Ontario: determination of prevalence and incidence using a validated administrative data algorithm**. *Diabetes Care* 2002, **25**(3):512-516.

13. Jette N, Reid AY, Quan H, Hill MD, Wiebe S: **How accurate is ICD coding for epilepsy?** *Epilepsia* 2010, **51**(1):62-69.

14. Rector TS, Wickstrom SL, Shah M, Thomas Greeenlee N, Rheault P, Rogowski J, Freedman V, Adams J, Escarce JJ: **Specificity and sensitivity of claims-based algorithms for identifying members of Medicare+Choice health plans that have chronic medical conditions**. *Health Serv Res* 2004, **39**(6 Pt 1):1839-1857.

15. Liu L, Allison JE, Herrinton LJ: **Validity of computerized diagnoses, procedures, and drugs for inflammatory bowel disease in a northern California managed care organization**. *Pharmacoepidemiol Drug Saf* 2009, **18**(11):1086-1093.

16. Sands BE, Duh M-S, Cali C, Ajene A, Bohn RL, Miller D, Cole JA, Cook SF, Walker AM: **Algorithms to identify colonic ischemia, complications of constipation and irritable bowel syndrome in medical claims data: development and validation**. *Pharmacoepidemiol Drug Saf* 2006, **15**(1):47-56.

17. Marrie RA, Fisk JD, Stadnyk KJ, Yu BN, Tremlett H, Wolfson C, Warren S, Bhan V: **The incidence and prevalence of multiple sclerosis in Nova Scotia, Canada**. *Can J Neurol Sci* 2013, **40**(6):824-831.

18. Austin PC, Daly PA, Tu JV: **A multicenter study of the coding accuracy of hospital discharge administrative data for patients admitted to cardiac care units in Ontario**. *Am Heart J* 2002, **144**(2):290-296.

19. Noyes K, Liu H, Holloway R, Dick AW: **Accuracy of Medicare claims data in identifying Parkinsonism cases: comparison with the Medicare current beneficiary survey**. *Mov Disord* 2007, **22**(4):509-514.

20. Fan J, Arruda-Olson AM, Leibson CL, Smith C, Liu G, Bailey KR, Kullo IJ: **Billing code algorithms to identify cases of peripheral artery disease from administrative data**. *J Am Med Inform Assoc* 2013, **20**(e2):e349-354.

21. Asgari MM, Wu JJ, Gelfand JM, Salman C, Curtis JR, Harrold LR, Herrinton LJ: **Validity of diagnostic codes and prevalence of psoriasis and psoriatic arthritis in a managed care population, 1996-2009**. *Pharmacoepidemiol Drug Saf* 2013, **22**(8):842-849.

22. Lurie N, Popkin M, Dysken M, Moscovice I, Finch M: **Accuracy of diagnoses of schizophrenia in Medicaid claims**. *Hosp Community Psychiatry* 1992, **43**(1):69-71.

23. Moscovice MF, Lurie N: **Minnesota: Plan Choice by the Mentally Ill in Medicaid Prepaid Health Plans**. *Adv Health Econ Health Serv Res* 1989, **10**:265-278.

24. Kokotailo RA, Hill MD: **Coding of stroke and stroke risk factors using international classification of diseases, revisions 9 and 10**. *Stroke* 2005, **36**(8):1776-1781.
